# Advances in Revista Brasileira de Medicina do Trabalho

**DOI:** 10.47626/1679-4435-2023-213

**Published:** 2023-11-24

**Authors:** Francisco Cortes Fernandes

**Affiliations:** President of Associação Nacional de Medicina do Trabalho

It is with great satisfaction that I write this editorial for the *Revista Brasileira de Medicina do Trabalho* (RBMT), my first as president of Associação Nacional de Medicina do Trabalho (the Brazilian Association of Occupational Medicine).

The journal had been hinting at the measures it would take toward improvement before its editor-in-chief, Dr. Mírian Parente, announced a series of editorial changes, including continuous (rolling) publication, reforming the editorial board, participation by foreign editors, editor training, and publishing pre-approved articles.^1^

In a subsequent editorial, “Evidence-based occupational health”,^2^ Dr. Wanderley Bernardo described training carried out in partnership with Associação Médica Brasileira (the Brazilian Medical Association) to develop guidelines, reflecting on the initiative’s success and enumerating the journal’s sources of pressure.

After the training sessions, evidence about clinical questions such as biological risk, heat, cold, mental health, vibration, sound, long COVID, remote work, and diagnosis was researched to produce recommendations.

In this issue, I present our guidelines on occupational diagnosis, with thanks to Dr. Bernardo for his partnership and guidance in their development. We are already working on the next set of guidelines, which will be out soon. We also present 20 articles by foreign and national authors, an impressive effort by the editors.

Now in its twenty-first year of publication, RBMT has a strong presence in the area. The demand for publication is great, which leads us to believe in the journal’s editorial success.

In another editorial, I provocatively questioned the need for this scientific journal, although after presenting the journal’s bibliometric data, its utility became clear.^3^ Obviously, the journal’s cost is high, given that it is bilingual, peer reviewed, and has a first-class editor. However, I am certain that these costs will pay off, since it can help us grow professionally, increasing our ability to research, write, and promote science. Our knowledge cannot be improved without discussion of theoretical and methodological aspects.

Finally, our conferences feature work by occupational physicians nationwide and abroad. This journal is a gateway for professionals to begin their scientific production, and they are encouraged to submit their articles. We are looking forward to everyone’s collaboration, since we have large research laboratories at our disposal that must not go to waste. Submit your work, and we hope to see everyone in Porto Alegre in November 2023.
